# Tanshinone IIA induces ferroptosis in gastric cancer cells through p53-mediated SLC7A11 down-regulation

**DOI:** 10.1042/BSR20201807

**Published:** 2020-08-18

**Authors:** Zhenhua Guan, Jing Chen, Xueliang Li, Na Dong

**Affiliations:** 1 Department of Gastroenterology, The First People’s Hospital of Lianyungang, Lianyungang 222000, Jiangsu Province, China; 2 Department of Gastroenterology, The First Affiliated Hospital of Nanjing Medical University, Nanjing 210029, Jiangsu Province, China; 3 Nursing Faculty, Hebei Women’s Vocational College, Shijiazhuang 050073, Hebei Province, China

**Keywords:** Ferroptosis, Gastric cancer, p53, ROS, Tanshinone IIA, xCT

## Abstract

Gastric cancer represents a malignant type of cancer worldwide. Tanshinone IIA (Tan IIA), a pharmacologically active component isolated from the rhizome of the Chinese herb *Salvia miltiorrhiza Bunge* (Danshen), has been reported to possess an anti-cancer effect in gastric cancer. However, its mechanisms are still not fully understood. In the present study, we found that Tan IIA induced ferroptosis in BGC-823 and NCI-H87 gastric cancer cells. Tan IIA increased lipid peroxidation and up-regulated Ptgs2 and Chac1 expression, two markers of ferroptosis. Ferrostatin-1 (Fer-1), an inhibitor of lipid peroxidation, inhibited Tan IIA caused-lipid peroxidation and Ptgs2 and Chac1 expression. In addition, Tan IIA also up-regulated p53 expression and down-regulated xCT expression. Tan IIA caused decreased intracellular glutathione (GSH) level and cysteine level and increased intracellular reactive oxygen species (ROS) level. p53 knockdown attenuated Tan IIA-induced lipid peroxidation and ferroptosis. Tan IIA also induced lipid peroxidation and ferroptosis in BGC-823 xenograft model, and the anti-cancer effect of Tan IIA was attenuated by Fer-1 *in vivo*. Therefore, Tan IIA could suppress the proliferation of gastric cancer via inducing p53 upregulation-mediated ferroptosis. Our study have identified a novel mechanism of Tan IIA against gastric cancer, and might provide a critical insight into the application of Tan IIA in gastric cancer intervention.

## Introduction

Gastric cancer is the fifth most common cancer in the world and the second leading cause of cancer deaths. Nearly 1 million new diagnoses are made every year. More than one-third of the world total occurs in China [1]. It is estimated that the 5-year survival rate of gastric cancer is lower than 20% in patients [2]. There are four defined subtypes of gastric cancer in the clinic, namely, papillary, mucinous, tubular, and signet ring cell cancers. The major risk factors of the occurrence and development of gastric carcinoma include gastroesophageal reflux disease, infection with *Helicobacter pylori*, dietary factors, and obesity [3]. Although the understanding of pathology of gastric cancer is increasing, conventional surgery, radiotherapy, and chemotherapy remain the primary therapeutic strategies [4].

Ferroptosis is a recently discovered type of regulated necrosis, which unlike apoptosis or necroptosis, is independent of caspase activity and receptor-interacting protein 1 (RIPK1) kinase activity. Instead, ferroptotic cells die following iron-dependent lipid peroxidation, a process which is antagonized by glutathione peroxidase 4 (GPX4) and ferroptosis suppressor protein 1 (FSP1) and lethal reactive oxygen species (ROS) derived from iron metabolism [5,6]. Recently, ferroptosis was found as a p53 (gene name: *TP53*)-mediated activity during tumor suppression. p53 can be recruited to the SLC7A11 promoter region to block the transcription of SLC7A11, which encodes xCT, a sodium-independent cystine-glutamate antiporter. xCT accounts for transportation of extracellular cystine into cells, and then cystine is reduced to cysteine used for glutathione (GSH) synthesis. p53 could induce ROS-mediated ferroptosis via xCT downregulation-caused decreased GSH production [7,8].

Tanshinone IIA (Tan IIA), a pharmacologically active component isolated from the rhizome of the Chinese herb *Salvia miltiorrhiza Bunge* (Danshen), has been clinically used in Asian countries for the prevention and treatment of coronary heart disease [9]. Recently, Tan IIA was found to exhibit antineoplastic effect in MKN-45 gastric cancer cells via inducing cell cycle arrest and apoptosis [10]. Tan IIA also inhibited the growth of gastric cancer AGS cells via suppressing epidermal growth factor receptor (EGFR), insulin like growth factor receptor (IGFR), vascular endothelial growth factor receptor (VEGFR) expression and blocking PI3K/Akt/mTOR, Ras/Raf/MEK/ERK pathways [11,12]. Tan IIA also possesses an effect on glucose metabolism in gastric cancer AGS cells [12]. Although these studies illustrated several mechanisms though which Tan IIA exerted its antineoplastic effect, the mechanisms are still not fully explored. What is of interest is Tan IIA up-regulated P53 in AGS and MKN-45 gastric cancer cells [10,13], which indicates that Tan IIA may induce ferroptosis in gastric cancer cells. In the present study, we employed BGC-823 and NCI-H87 gastric cancer cells to investigate whether Tan IIA could induce ferroptosis and its underlying mechanisms.

## Materials and methods

### Materials

Tan IIA (S2365) and ferrostatin-1 (Fer-1, S7245) were purchased from Selleckchem Company (Shanghai, China). Anti-p53 (ab26), anti-xCT (ab175186), anti-β-actin (ab8226), goat anti-rabbit-HRP (ab6721), goat anti-mouse-HRP (ab6728) were obtained from Abcam Company (Cambridge, MA).

### Cell line

BGC-823 and NCI-H87 gastric cancer cells was obtained from American Type Culture Collection (ATCC) and cultured in RPMI-1640 medium containing 10% fetal bovine serum (FBS), 1% penicillin (100 U/ml), and streptomycin (100 U/ml). The cells were maintained at 37°C, in a humidified incubator with 5% CO_2_ and in mid-log phase were used.

### Cell viability assay

Cell viability was assessed by CellTiter-Lumi™ Luminescent Cell Viability Assay Kit (C0065M) from Beyotime Biotech Company (Nanjing, China). Briefly, 100 μl medium per well containing 5000 BGC-823 or NCI-H87 gastric cancer cells were seeded into 96-well plates and treated with 100, 33.3, 11.1, 3.7, 1.2, 0.4, 0.13, 0.046 μM Tan IIA. The plates were incubated at 37°C in a 5% CO_2_ incubator for 72 h. After treatment, 100 μl Cell Titer-Lumi™ reagent was added into each well and kept at room temperature for 10 min. Luminescence signals were then detected using a Biotek synergy H1 microplate reader. Percentage of cell viability (%) = (test group − negative control)/(control group − negative control) × 100%. IC_50_ was calculated using GraphPad Prism 6.0 software.

### Cell death assay

Cell death was detected by 7-amino-actinomycin D (7-AAD) (559925) from BD Biosciences. Briefly, 2 × 10^4^ BGC-823 or NCI-H87 gastric cancer cells were seeded into 12-well plates and treated with Tan IIA or Tan IIA combined with Fer-1 for 72 h. At the end of treatment, cells were harvested and stained with 7-AAD as described by the manufacturer’s instructions. Percentage of cell death were assessed by flow cytometry using an Accuri 6 cytometer (BD Biosciences).

### Measurement of intracellular GSH

Intracellular GSH was measured using the DTNB-GSSH reductase recycling assay kit (Beyotime Biotechnology, Nanjing, China) as described by manufacturer’s protocol. Briefly, 20 mg tumor tissue was digested using trypsin into single cell, the collected digested cells or BGC-823/NCI-H87 gastric cancer cells were added into protein-removing buffer S, homogenized on ice with a homogenizer, and centrifuged at 10000×***g*** for 10 min at 4°C to get the supernatant used for intracellular total GSH assay. GSH content was expressed as a ratio to the absorbance value at 412 nm of the control cells.

### Measurement of intracellular cysteine

Intracellular cysteine was measured by using a cysteine assay kit (Nanjing Jiancheng Bioengineering Institute, Nanjing, China) according to the manufacturer’s protocol. Briefly, 20 mg tumor tissue was digested using trypsin into single cell, the collected digested cells or BGC-823 or NCI-H87 gastric cancer cells were added into reagent A, homogenized on ice, and centrifuged at 8000×***g*** for 4 min at 4°C to obtain the supernatant for assay. After the protein concentration was measured, 20 µl sample was incubated with 100 µl reagent B and 100 µl reagent C for 15 min at room temperature and read at absorbance 600 nm in a microplate reader. Finally, the results were expressed as a ratio to the absorbance value of the control cells.

### Detection of intracellular ROS

A total of 20 mg tumor tissue was digested using trypsin into single cell, ROS level in the collected digested cells or BGC-823 or NCI-H87 gastric cancer cells was determined using an oxidation sensitive dye, 2′,7′-dichlorofluorescein diacetate (DCFH-DA) (Sigma, St. Louis, Missouri, U.S.A.). Cells were incubated with 50 μM dye for 30 min and washed with PBS. Fluorescence value was measured by using a fluorescence microplate reader (Molecular Devices, U.S.A.) at excitation and emission wavelengths of 485 and 530 nm, respectively. Relative fluorescence unit (RFU) was the difference in fluorescence values obtained at time 0 and 5 min. The fold change of RFU was compared with control group.

### RNA interference

BGC-823 or NCI-H87 gastric cancer cells were seeded in six-well plates (2 × 10^5^) and for TP53 siRNA treatment, the cells were transfected with 20 μM TP53 siRNA (GenePharma, Shanghai, China). The siRNA target sequence was prepared as follows: 5′-CACCUCACUGCAUGGACGAUCUGUU-3′. The transfection was performed using Lipofectamine 3000 (Invitrogen, Carlsbad, CA, U.S.A.) in Opti-MEM medium (Gibco BRL, Grand Island, NY, U.S.A.) for 4 h. Then, the medium was replaced with fresh RPMI-1640 medium containing 10% FBS. After another 24-h culture, cells were harvested, RNA samples were prepared for analyzing TP53 mRNA expression using RT-qPCR and protein samples were prepared for analyzing p53 protein expression using Western Blot.

### Lipid peroxidation

A total of 20 mg tumor tissue was digested using trypsin into single cell. Lipid peroxidation in digested cells and BGC-823/NCI-H87 gastric cancer cells was evaluated by BODIPY™ 581/591 C11 dye from BD Biosciences. Briefly, 2 × 10^4^ cells were seeded into 12-well plates and treated with Tan IIA or Tan IIA combined with Fer-1 for 72 h. At the end of treatment, cells were harvested and stained with BODIPY™ 581/591 C11 dye as described by the manufacturer’s instructions. Lipid peroxidation was analyzed by flow cytometry using an Accuri 6 cytometer (BD Biosciences). The fold change of lipid peroxidation = test group ratio/mean of control group ratio.

### Fluorescence-quantitative reverse transcription-polymerase chain reaction (RT-qPCR)

After treatment, total RNA was isolated from BGC-823 or NCI-H87 gastric cancer cells and tumor tissue using RNA isolater Total RNA Extraction Reagent (Vazyme Biotech Co. Ltd) and reverse transcribed into cDNA using HiScript II Q Select RT SuperMix for qPCR (Vazyme Biotech Co. Ltd) according to the supplier’s protocol. Real-time PCR was performed with gene-specific primers (Table 1, Thermo Fisher Scientific Inc.) with 18s as internal control. The reactions were performed in an ABI Prism® 7900 High-Throughput Real-Time PCR System (Applied Biosystems, Foster City, CA, U.S.A.). The comparative threshold cycle (*C*_T_) method was used for relative quantification of target gene expression, which was plotted as fold of control.

**Table 1:** Primer sequences in quantitative RT-qPCR

Gene	Sequences
*18s*	Forward: ACCCGTTGAACCCCATTCGTGA
	Reverse: GCCTCACTAAACCATCCAATCGG
*hPtgs2*	Forward: CGGTGAAACTCTGGCTAGACAG
	Reverse: GCAAACCGTAGATGCTCAGGGA
*hChac1*	Forward: GTGGTGACGCTCCTTGAAGATC
	Reverse: GAAGGTGACCTCCTTGGTATCG
*TP53*	Forward: TGCCGAGACTGATAGCTGAG
	Reverse: AAAACTTCAAAGTGGGGTTA
SLC7A11	Forward: TCCTGCTTTGGCTCCATGAACG
	Reverse: AGAGGAGTGTGCTTGCGGACAT

### Western blot

Total proteins were obtained using radio-immunoprecipitation assay (RIPA) buffer (Beyotime, Shanghai, China) supplemented with the protease inhibitor cocktail (Sigma–Aldrich). In certain experiments, cytoplasmic and mitochondrial proteins were extracted using Cell Mitochondria Isolation Kit according to the manufacturer’s instructions, nuclear and cytoplasmic proteins were extracted using Nuclear and Cytoplasmic Protein Extraction Kit (Beyotime) according to the manufacturer’s instructions. Protein concentrations were measured using BCA protein assay kit (Beyotime). The protein samples were separated by SDS/PAGE and blotted on to polyvinylidene fluoride (PVDF) membranes (Millipore, Billerica, MA, U.S.A.). Next, the membranes were incubated at 4°C overnight with primary antibodies against p53 (ab26), xCT (ab175186), β-actin (ab8226), followed by HRP-conjugated secondary antibodies. The immunoblot signals were visualized by enhanced chemiluminescence detection kit (Amersham Pharmacia Biotec, Buckinghamshire, U.K.). The gray-scale values were quantified by ImageJ software (NIH, Bethesda, MD, U.S.A.).

### *In vivo* experiments

All experiments were approved by the Animal Ethics Committee of The First People’s Hospital of Lianyungang (ethic approval number: 2019-11-007) and were carried out at The First People’s Hospital of Lianyungang following the guidelines in the Guide for the Care and Use of Laboratory Animals published by the National Institutes of Health. NOD-SCID (NOD CB17-Prkdcscid/NcrCrl, male, 5 weeks of age) mice were obtained from Beijing Vital River Laboratory Animal Technology Co., Ltd (Beijing, China). All mice were housed under a setting of 12-h light/dark cycle at 22 ± 1°C, 55% humidity and fed with water and food provided at regular time. During the entire maintenance period, all mice were permitted free cage activity without joint immobilization. The initial body weights of the mice were between 20 and 23 grams. After subcutaneous injection of 2 × 10^6^ BGC-823 gastric cancer cells into the back of NOD-SCID mice, the mice were treated with or without Tan IIA (50 mg/kg) or Tan IIA in combination with Fer-1 (50 mg/kg). Tan IIA was diluted in DMSO:Methanol:Hydroxypropyl-β-cydodextrin (HP-β-CD) = 1:1:1. Fer-1 was also dissolved in DMSO:Methanol:HP-β-CD. Seven days after BGC-823 gastric cancer cells injection, intraperitoneal injection with Tan IIA was carried out every other day followed by killing at day 22 of tumor cell inoculation. All mice were killed by dislocation of the cervical vertebrae. Before killing, the tumor volume was measured every 3 days. All experiments were carried out using six mice each group in three independent experiments of a time-dependent manner with three time points.

### Statistical analysis

All data represent at least three independent experiments and are expressed as mean ± SD. Statistical comparisons were made using one-way ANOVA. *P*-values of less than 0.05 were considered to represent statistical significance.

## Results

### Tan IIA induces cell death in BGC-823 and NCI-H87 gastric cancer cells

**Figure 1:**
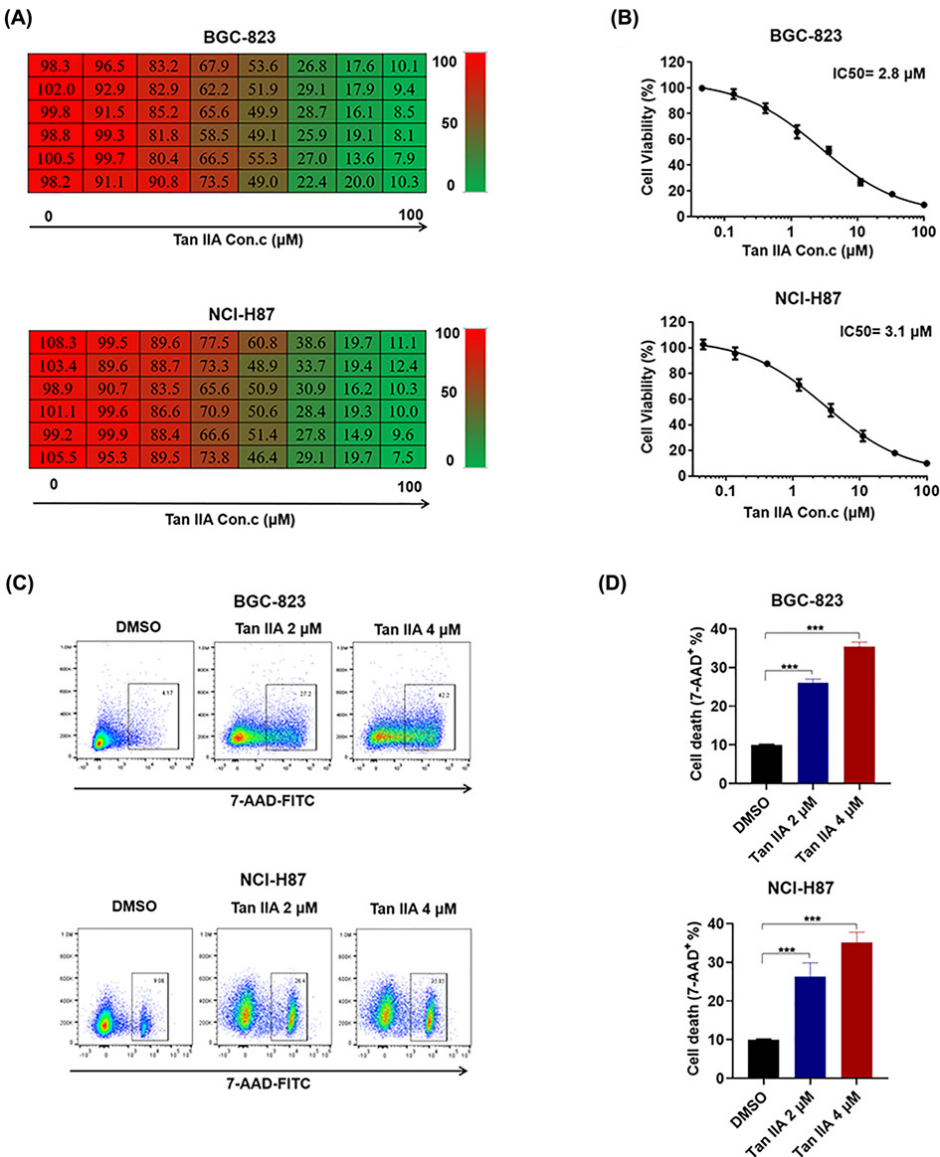
Tan IIA inhibits cell growth and induces cell death in BGC-823 and NCI-H87 gastric cancer cells (**A**) BGC-823 and NCI-H87 cells were treated with serially diluted (1:3) concentrations of Tan IIA with the highest concentration of 10 μM for 72 h. Cell viability was detected by MTT. (**B**) IC_50_ of Tan IIA on BGC-823 and NCI-H87 cell growth. (**C**) BGC-823 and NCI-H87 cells were treated with 2 and 4 μM Tan IIA for 72 h, cells were stained with 7-AAD and cell death was assessed by flow cytometry. (**D**) Histogram statistics of cell death in (**C**). Data are shown as the mean ± SD (*n*=3). ***P<0.001 vs control group.

To investigate whether Tan IIA possesses an anti-cancer effect, we employed MTT assay to test the effect of Tan IIA on the cell viability of BGC-823 and NCI-H87 gastric cancer cells. When BGC-823 and NCI-H87 cells were treated with serially diluted (1:3) concentrations of Tan IIA with the highest concentration of 10 μM, cell viability was inhibited by Tan IIA in a concentration-dependent manner (Figure 1A). The IC_50_ of Tan IIA in BGC-823 and NCI-H87 cells was accounted according to this set of data, and it was 2.8 μM in BGC-823 cells and 3.1 μM in NCI-H87 cells (Figure 1B). In addition, flow cytometry was performed to detect cell death using 7-AAD staining. According to flow cytometry result, Tan IIA induced cell death in a concentration-dependent manner in BGC-823 and NCI-H87 cells (Figure 1D) and representative pictures of flow cytometry was shown in Figure 1C. Taken together, these results shows that Tan IIA could induce cell death in BGC-823 and NCI-H87 gastric cancer cells.

### Tan IIA induces ferroptosis in BGC-823 and NCI-H87 gastric cancer cells

**Figure 2:**
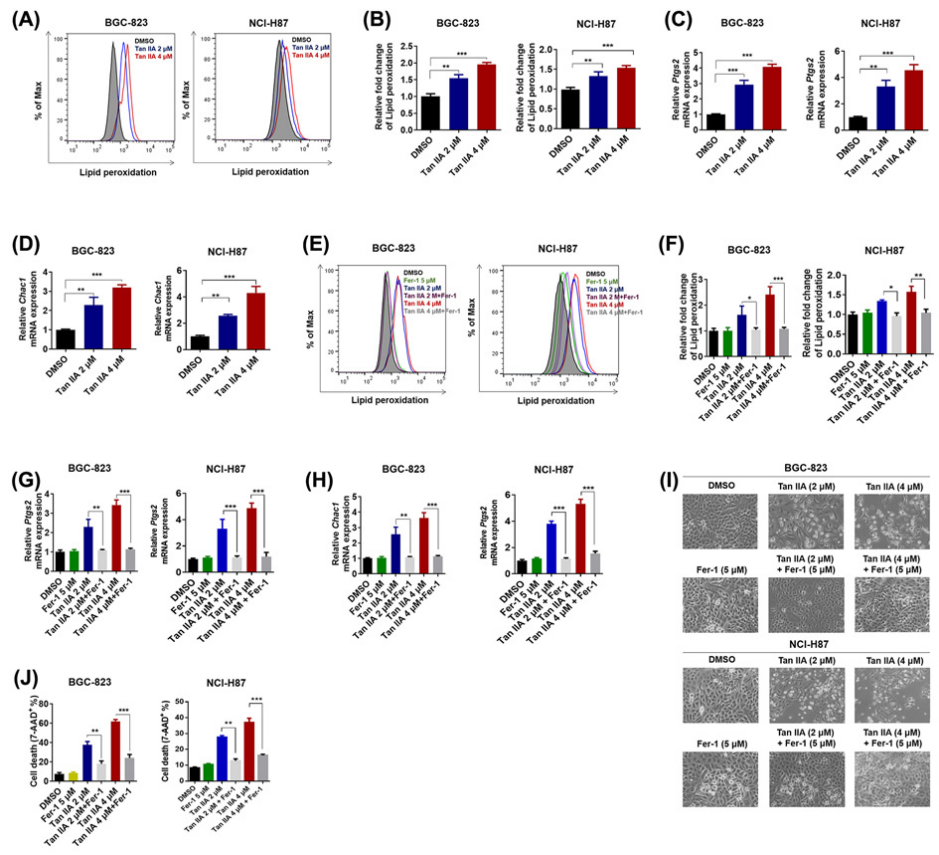
Tan IIA induces ferroptosis in BGC-823 and NCI-H87 gastric cancer cells (**A**) BGC-823 and™ NCI-H87 cells were treated with 2 and 4 μM Tan IIA for 72 h, cells were stained with BODIPY™ 581/591 C11 dye and lipid peroxidation was detected by flow cytometry. (**A**) Histogram statistics of cell death in (**B**). (**C**,**D**) BGC-823 cells were treated with Tan IIA as mentioned above. The expression of two marker genes of ferroptosis, *Ptgs2* and *Chac1* was detected by RT-qPCR. (**E**) BGC-823 and NCI-H87 cells were treated with 2 and 4 μM Tan IIA or Tan IIA combined with 5 μM Fer-1 for 72 h, cells were stained with BODIPY™ 581/591 C11 dye and lipid peroxidation was detected by flow cytometry. (**F**) Histogram statistics of cell death in (**E**). (**G**,**H**) BGC-823 and NCI-H87 cells were treated as mentioned in (**E**). The expression of two marker genes of ferroptosis, *Ptgs2* and *Chac1* was detected by RT-qPCR. (**I**) Representative pictures of BGC-823 and NCI-H87 cells post Tan IIA or Tan IIA combined with 5 μM Fer-1 treatment. (**J**) BGC-823 and NCI-H87 cells were treated as mentioned in (**E**), cells were stained with 7-AAD and cell death was assessed by flow cytometry. Data are shown as the mean ± SD (*n*=3). *P<0.05 vs control group; **P<0.01 vs control group; ***P<0.001 vs control group.

Ferroptosis is a recently discovered type of regulated necrosis. Iron-dependent lipid peroxidation is the specific process of ferroptosis. To investigate whether Tan IIA could induce ferroptosis in BGC-823 and NCI-H87 gastric cancer cells, BGC-823 and NCI-H87 cells were treated with 2 and 4 μM Tan IIA for 72 h and lipid peroxidation was detected by flow cytometry. The result showed that lipid peroxidation was increased by both 2 and 4 μM Tan IIA in BGC-823 and NCI-H87 gastric cancer cells (Figure 2A,B). The expression of another two marker genes of ferroptosis, *Ptgs2* and *Chac1*, was also significantly up-regulated by Tan IIA (Figure 2C,D). Moreover, when BGC-823 and NCI-H87 gastric cancer cells were treated with Tan IIA combined with 5 μM Fer-1, which could specifically inhibit oxidative lipid damage [14], lipid peroxidative induced by Tan IIA was significantly decreased (Figure 2E,F). The increased expression of Ptgs2 and Chac1 induced by Tan IIA was also abrogated by Fer-1 (Figure 2G,H). Strikingly, the morphological change of BGC-823 and NCI-H87 cells induced by Tan IIA was also prevented by Fer-1 (Figure 2I). In addition, the cell death caused by Tan IIA was deservedly attenuated by Fer-1 (Figure 2J). Taken together, these results suggest that Tan IIA causes cell death of BGC-823 and NCI-H87 gastric cancer cells via inducing ferroptosis.

### Tan IIA induces ferroptosis via up-regulation of p53 expression

**Figure 3:**
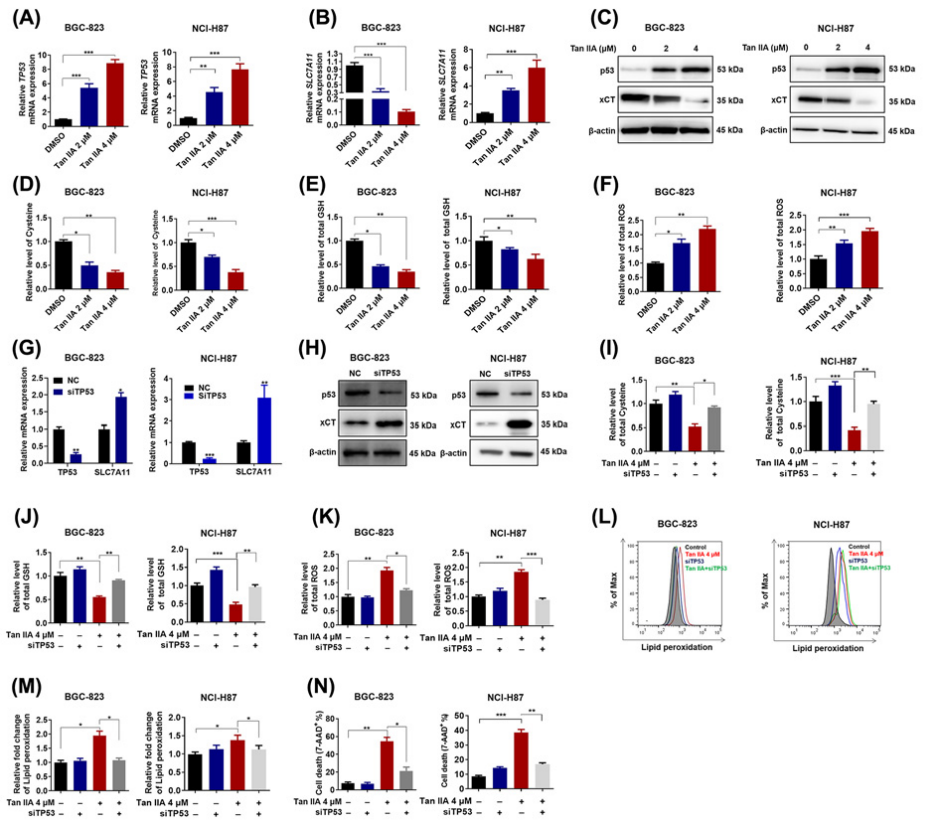
p53 mediates Tan IIA-induced ferroptosis in BGC-823 and NCI-H87 gastric cancer cells (**A**,**B**) BGC-823 and NCI-H87 cells were treated with 2 and 4 μM Tan IIA for 72 h, the mRNA expression of TP53 and its target gene *SLC7A11* was detected by RT-qPCR. (**C**) BGC-823 and NCI-H87 cells were treated with Tan IIA as mentioned above. The protein expression of p53 and xCT was detected by Western Blot. (**D**–**F**) BGC-823 and NCI-H87 cells were treated with Tan IIA as mentioned above. Intracellular cysteine level, GSH level and ROS level were measured. (**G**,**H**) BGC-823 and NCI-H87 cells were transfected with 20 μM TP53 siRNA and negative control (NC) sequence using Lipofectamine 3000 for 4 h, after 24 h more culture with fresh RPMI-1640 medium with 10% FBS, cells were harvested. The knockdown efficiency of TP53 and the expression of its target gene *SLC7A11* in both BGC-823 and NCI-H87 gastric cancer cells was detected by RT-qPCR and Western Blot. (**I**–**K**) BGC-823 and NCI-H87 cells were pretreated with TP53 siRNA, then the cells were treated with Tan IIA for 72 h, intracellular cysteine level, GSH level and ROS level were measured. (**L–N**) BGC-823 and NCI-H87 cells were treated with TP53 siRNA and Tan IIA as mentioned above. Then lipid peroxidation and cell death was detected by flow cytometry. Data are shown as the mean ± SD (*n*=3). *P<0.05 vs control group; **P<0.01 vs control group; ***P<0.001 vs control group.

p53 plays a critical role in ferroptosis [7]. It was reported that Tan IIA up-regulated p53 in AGS and MKN-45 gastric cancer cells [10,13]. Thus, we hypothesized that Tan IIA induced ferroptosis via up-regulation of p53 expression in BGC-823 and NCI-H87 gastric cancer cells. We first checked the influence of Tan IIA on the expression of TP53. As shown in Figure 3A, Tan IIA increased TP53 mRNA expression as expected. SLC7A11, a target gene of p53 and the key transporter for cysteine import, was significantly down-regulated by Tan IIA at mRNA level (Figure 3C). Consistently, Tan IIA also up-regulated TP5 (p53) expression and down-regulated xCT (SLC7A11) expression at protein level (Figure 3C). Moreover, intracellular cysteine level and GSH was decreased after Tan IIA treatment (Figure 3D,E). In contrast, intracellular ROS level was increased after Tan IIA treatment in both BGC-823 and NCI-H87 gastric cancer cells (Figure 3F). In order to clarify the role of p53 in Tan IIA-induced ferroptosis, knockdown of TP53 using siRNA was performed. The knockdown efficiency at mRNA level is shown in Figure 3G and the knockdown efficiency at protein level is shown in Figure 3H. After knockdown of TP53, SLC7A11 gene expression at both mRNA and protein was increased (Figure 3G,H). In addition, decreased intracellular cysteine and GSH level by Tan IIA treatment were both reverted by TP53 knockdown (Figure 3I,J). Increased intracellular ROS was inversely inhibited by TP53 knockdown (Figure 3K). Thus, we next tested whether TP53 knockdown could attenuate Tan IIA-induced ferroptosis. As shown in Figure 3L,M, the lipid peroxidation by Tan IIA was inhibited by TP53 knockdown in both BGC-823 and NCI-H87 gastric cancer cells. Besides, Tan IIA-induced cell death was also abrogated by TP53 knockdown (Figure 3N). Taken together, these results suggest Tan IIA induces ferroptosis via up-regulation of p53 expression.

### Tan IIA suppresses tumor growth and induces ferroptosis *in vivo*

**Figure 4:**
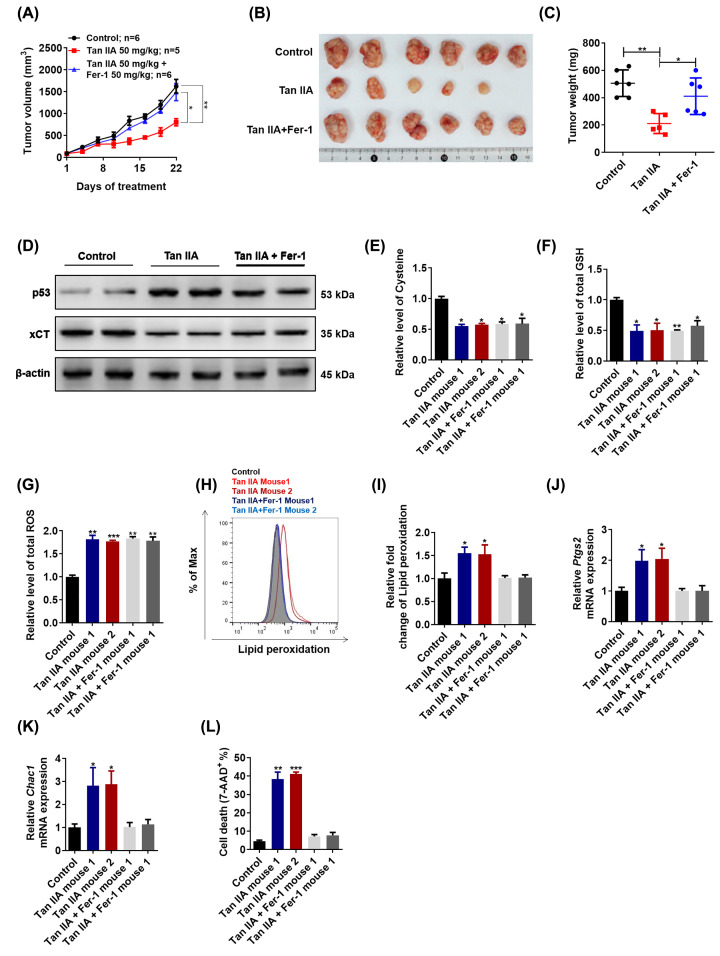
Tan IIA suppresses tumor growth and induces ferroptosis *in vivo* (**A**) Mice were treated with Tan IIA (50 mg/kg), Fer-1 (50 mg/kg) and Tan IIA (50 mg/kg) combined with Fer-1 (50 mg/kg) for 3 weeks, tumor volume was measured every 3 days (*n*=5–6). (**B, C**) After treatment with Tan IIA (50 mg/kg), Fer-1 (50 mg/kg) and Tan IIA (50 mg/kg) combined with Fer-1 (50 mg/kg) for 3 weeks, tumors in these groups were removed, tumor pictures were captured (**B**) and tumors were weighed (**C**). (**D**) A total of 20 mg tumor tissue in two mice of each group was isolated. The expression of p53 and xCT was detected by Western Blot and RT-qPCR. (**E**–**G**) A total of 20 mg tumor tissue in two mice of each group was isolated, intracellular cysteine level, GSH level and ROS level were measured. (**H, I**) A total of 20 mg tumor tissue in two mice of each group was isolated and digested into single cell. Then the cells of each group were stained with BODIPY™ 581/591 C11 and lipid peroxidation was analyzed by flow cytometry. (**J**,** K**) A total of 20 mg tumor tissue in two mice of each group was isolated and total RNA sample was extracted. The expression of two marker genes, *Ptgs2* and *Chac1* was detected by RT-qPCR. (**L**) A total of 20 mg tumor tissue in two mice of each group was isolated and digested into single cell. Then the cells of each group were stained with 7-AAD and cell death was analyzed by flow cytometry. Data are shown as the mean ± SD (*n*=3). *P<0.05 vs control group; **P<0.01 vs control group; ***P<0.001 vs control group.

To investigate the anti-tumor effect of Tan IIA, as well as the role of ferroptosis played in the anti-tumor effect of Tan IIA, BGC-823 cells xenograft model was employed. After treatment with 50 mg/kg Tan IIA or 50 mg/kg Tan IIA combined with 50 mg/kg Fer-1 for 3 weeks, the results showed that Tan IIA also possessed obvious anti-tumor effect *in vivo.* However, the anti-tumor effect of Tan IIA was attenuated by Fer-1 to a great extent (Figure 4A-C). The p53 expression was up-regulated while the xCT expression was down-regulated by Tan IIA, which was consistent with *in vitro* results, and Fer-1 had no obvious effect on p53 and xCT expression (Figure 4D). Consistently, Tan IIA decreased intracellular cysteine level, GSH level and increased intracellular ROS level while Fer-1 has no effect on them (Figure 4E–G). Moreover, Tan IIA expectedly induced lipid peroxidation *in vivo* as Tan IIA did *in vitro*, while Fer-1 significantly suppressed it (Figure 4H, I). The expression of two marker genes of ferroptosis, Ptgs2 and Chac1 was increased by Tan IIA treatment and Fer-1 significantly abrogated it, which indicated that Tan IIA induced ferroptosis *in vivo* and Fer-1 abrogated it (Figure 4J, K). In addition, Tan IIA induced cell death *in vivo*, which was also attenuated by Fer-1 (Figure 4L). Taken together, these results suggest that Tan IIA exerts anti-tumor effect via inducing ferroptosis *in vivo*.

## Discussion

Our present study employed BGC-823, NCI-H87 gastric cancer cells and xenograft model to test the anti-cancer effect of Tan IIA *in vitro* and *in vivo*, and identified a novel mechanism that Tan IIA induced ferroptosis via p53-mediated SLC7A11 down-regulation. This might provide a critical insight into the application of Tan IIA in gastric cancer intervention.

Ferroptosis has been established as a non-apoptotic regulated cell death. The key feature of ferroptosis is iron-dependent lipid peroxidation. ROS plays a key role in lipid peroxidation, thus factors which could change intracellular ROS level might also induce ferroptosis [15]. GSH is an important antioxidant for maintaining redox homeostasis [16]. Any factors increasing GSH consume or disrupting GSH synthesis could lead to increased intracellular ROS level and then induce ferroptosis. We first found that Tan IIA could induce ferroptosis in BGC-823 and NCI-H87 gastric cancer cells which was characterized by increased lipid peroxidation and Ptgs2, Chac1 expression. The inducible death of ferroptosis could be prevented by lipid peroxidation inhibitors [17,18]. Fer-1, a lipid peroxidation inhibitor, significantly attenuated Tan IIA caused increased lipid peroxidation and Ptgs2, Chac1 expression, which indicated that Tan IIA indeed induced ferroptosis in BGC-823 and NCI-H87 gastric cancer cells. Then, we found that Tan IIA could up-regulate the p53 expression and inhibit the expression of its target gene *SLC7A11* in BGC-823 and NCI-H87 gastric cancer cells. SLC7A11 encodes the cystine transporter xCT for cystine uptake, which could be reduced to cysteine used for GSH synthesis. Depletion of GSH via inhibition of xCT was also found to be an efficient pathway leading to ferroptosis in cancer cells [17]. Consistently, Tan IIA indeed decreased intracellular cysteine level, GSH level and increased ROS level in BGC-823 and NCI-H87 gastric cancer cells. Knockdown of p53 was able to abrogate Tan IIA-induced ferroptosis and attenuated the Tan IIA-caused cell death. These findings suggest Tan IIA induces ferroptosis via p53-mediated SLC7A11 down-regulation. In addition, these findings was also be verified in BGC-823 gastric cancer cells xenograft model. Fer-1 was able to decrease the anti-tumor effect of Tan IIA *in vivo*, which indicated an important role of ferroptosis played in the anti-tumor effect of Tan IIA.

## Conclusion

In conclusion, we demonstrated in the present study that ferroptosis is a pathway leading to Tan IIA-induced BGC-823 and NCI-H87 gastric cancer cell death. Tan IIA induces ferroptosis via p53-mediated SLC7A11 down-regulation. Our study might provide a critical insight into the application of Tan IIA in gastric cancer intervention.

## Abbreviations

7-AAD: 7-amino-actinomycin D
FBS: fetal bovine serum
Fer-1: ferrostatin-1
GSH: glutathione
HP-β-CD: hydroxypropyl-β-cydodextrin
RFU: relative fluorescence unit
ROS: reactive oxygen species
Tan IIA: Tanshinone IIA
IGFR: insulin like growth factor receptor
VEGFR: vascular endothelial growth factor receptor
HRP: horse radish peroxidase
EGFR: epidermal growth-factor receptor
RT-qPCR: fluorescence-quantitative reverse transcription-polymerase chain reaction
